# Correction to: Understanding Diversity in the Meaning of Cohabitation Across Europe

**DOI:** 10.1007/s10680-023-09688-x

**Published:** 2023-12-20

**Authors:** Nicole Hiekel, Aart C. Liefbroer, Anne-Rigt Portman

**Affiliations:** 1 Netherlands Interdisciplinary Demographic Institute (NIDI), University of Groningen (RUG), Lange Houtstraat 19, 2518 CV The Hague, The Netherlands; 2 Netherlands Interdisciplinary Demographic Institute/University Medical Center Groningen and VU University Amsterdam, De Boelelaan 1081, 1081 HV Amsterdam, The Netherlands; 3 Department of Sociology, Utrecht University, The Netherlands, Padualaan 14, 3584 CH Utrecht, The Netherlands

## Correction to: European Journal of Population https://doi.org/10.1007/s10680-014-9321-1

Cross-national research comparing unmarried cohabitation across contexts that vary in its societal diffusion has tended to assign one predominant meaning to cohabitation for a whole country. Overlooking the heterogeneity between individuals in cohabiting unions leads to findings that challenge the validity of these typologies. Hiekel, Liefbroer, and Poortman (2014) propose a typology of meanings of cohabitation based on how cohabiters currently view their union, as defined by self-reported intentions to marry and attitudes toward the institution of marriage.

In Table 2, Hiekel et al. present the empirical classification of cohabiters into different types of cohabitation based on the response patterns to three indicators: marital intentions, attitudes toward the institution of marriage, and perceived economic deprivation. The text on page 400 correctly states that the classification as “conformist” is based on positive intentions to marry as well as agreeing that marriage is an outdated institution, while perceived economic deprivation is not considered. In the said table, however, the indicator for the marital attitude is set on “No,” while “Yes” would be correct. In sum, while the reported value on the variable in the table is erroneous, the description of this type’s operationalization presented in the text accompanying the table is correct. This erratum therefore contains a corrected version of Table 2 of the original publication of the Hiekel et al. (2014) study.

**Table 2:** An empirical typology of different meanings of cohabitation based on three indicators

	Intends to marry within three years	Agrees that marriage is outdated	Has trouble making ends meet
Prelude to marriage	Yes	No	Not used^a^
Trial marriage	No	No	No
Economic reasons	No	No	Yes
Conformist	Yes	Yes	Not used^a^
Refusal of marriage	No	Yes	Not used^a^
Marriage is irrelevant	No	Neutral	Not used^a^

^a^This indicator is not used to classify respondents in that type of cohabitation

In Table 3 on page 400, Hiekel et al. present the percent distribution of different meanings of cohabitation including an entropy measure of variation for n = 10 countries and n = 9,113 cohabiters. The formula to obtain the entropy measure is correct and can be found in footnote 1 on page 403. The reported results on the entropy measure in the said table are not correct. The text that reports and interprets the results on page 403 refers to the numbers of the correct calculation of the entropy measures. In sum, while the reported numbers of the entropy measures in the table are erroneous, the conclusions drawn and presented in the text accompanying the table are correct.

**Table 3:** Percent (weighted) distribution of different meanings of cohabitation and measure of variation (entropy), by country (n = 9,113)

	Western and Northern Europe					Central and Eastern Europe				
	Austria^a^	Germany	France	Norway	Bulgaria	Georgia	Hungary	Lithuania	Romania	Russia
Prelude to marriage	27.0	20.2	24.8	10.5	16.0	66.0	24.2	23.2	39.0	25.9
Trial marriage	15.2	26.4	19.3	29.3	1.7	5.7	13.4	8.1	5.2	9.8
Economic reasons	1.2	2.2	6.8	0.9	9.6	10.5	2.6	2.4	5.2	16.9
Conformist	17.9	21.0	21.5	20.5	32.3	2.1	19.3	13.6	4.8	14.5
Refusal of marriage	18.3	17.1	15.4	31.9	13.7	1.9	12.7	14.2	10.7	11.6
Marriage is irrelevant	20.5	13.0	12.3	6.9	26.7	13.7	27.9	38.7	35.0	21.3
Proportion cohabiting of all co-resident unions	30.2	12.2	19.7	23.0	10.8	14.2	13.6	11.4	5.3	15.4
Measure of variation of cohabitation types (entropy)	0.71	0.72	0.75	0.66	0.69	0.48	0.71	0.67	0.62	0.75
Total *n*	879	748	1,173	1,571	840	906	1,148	562	449	873

Table includes weighted percentages and unweighted number of cases

^a^Different age range (18–45 years)

This erratum therefore contains a corrected version of Table 3 of the original publication of the Hiekel et al. (2014) study.

